# Bifunctional Manipulation of Terahertz Waves with High‐Efficiency Transmissive Dielectric Metasurfaces

**DOI:** 10.1002/advs.202205499

**Published:** 2022-12-09

**Authors:** Zhuo Wang, Yao Yao, Weikang Pan, Haoyang Zhou, Yizhen Chen, Jing Lin, Jiaming Hao, Shiyi Xiao, Qiong He, Shulin Sun, Lei Zhou

**Affiliations:** ^1^ State Key Laboratory of Surface Physics and Key Laboratory of Micro and Nano Photonic Structures (Ministry of Education) Fudan University Shanghai 200433 P. R. China; ^2^ Shanghai Engineering Research Centre of Ultra Precision Optical Manufacturing Department of Optical Science and Engineering School of Information Science and Technology Fudan University Shanghai 200433 P. R. China; ^3^ Institute of optoelectronics Fudan University Shanghai 200433 P. R. China; ^4^ Shanghai Institute for Advanced Communication and Data Science Shanghai University Shanghai 200444 P. R. China; ^5^ Yiwu Research Institute of Fudan University Chengbei Road Yiwu City Zhejiang 322000 P. R. China

**Keywords:** circular polarization, dielectric, spin‐multiplexed metasurface, transmission configuration, wavefront controls

## Abstract

Multifunctional terahertz (THz) devices in transmission mode are highly desired in integration‐optics applications, but conventional devices are bulky in size and inefficient. While ultra‐thin multifunctional THz devices are recently demonstrated based on reflective metasurfaces, their transmissive counterparts suffer from severe limitations in efficiency and functionality. Here, based on high aspect‐ratio silicon micropillars exhibiting wide transmission‐phase tuning ranges with high transmission‐amplitudes, a set of dielectric metasurfaces is designed and fabricated to achieve efficient spin‐multiplexed wavefront controls on THz waves. As a benchmark test, the photonic‐spin‐Hall‐effect is experimentally demonstrated with a record high absolute efficiency of 92% using a dielectric metasurface encoded with geometric phases only. Next, spin‐multiplexed controls on circularly polarized THz beams (e.g., anomalous refraction and focusing) are experimentally demonstrated with experimental efficiency reaching 88%, based on a dielectric meta‐device encoded with both spin‐independent resonant phases and spin‐dependent geometric phases. Finally, high‐efficiency spin‐multiplexed dual holographic images are experimentally realized with the third meta‐device encoded with both resonant and geometric phases. Both near‐field and far‐field measurements are performed to characterize these devices, yielding results in agreement with full‐wave simulations. The study paves the way to realize multifunctional, high‐performance, and ultra‐compact THz devices for applications in biology sensing, communications, and so on.

## Graphical abstract

High‐quality dielectric metasurfaces are experimentally demonstrated to achieve efficient spin‐multiplexed wavefront controls on transmissive terahertz lights, including photonic spin Hall effect, anomalous refraction and focusing, and holographic dual‐images. The adopted high aspect‐ratio silicon meta‐atoms exhibit high transmission, large phase accumulation, and desired cross‐polarization conversion ability, simultaneously. This work can stimulate realization of many high‐efficiency, ultra‐compact, and multifunctional terahertz devices.

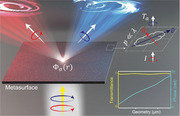

## 1 Introduction

The terahertz (THz) frequency regime, located in‐between microwave and optical frequency ranges, has attracted intensive attention recently, stimulated by many applications such as information communication, biological and chemical detection, medical health, security, etc.^[^ ^1^, ^2^ ^]^ However, conventional THz devices made by naturally existing dielectric materials are usually of bulky sizes, low efficiencies, and limited wave‐control functionalities, which hinder their applications in THz technologies.^[^ ^3^, ^4^, ^5^ ^]^ These shortcomings are ultimately caused by weak interactions between THz light and conventional materials in this frequency domain. High‐efficiency, ultra‐compact, and multifunctional wave‐manipulation devices are highly desired for future integration‐optics applications in the THz regime.

Metasurfaces, composed of subwavelength planar microstructures (i.e., meta‐atoms) with pre‐designed optical responses arranged in certain global sequences, have exhibited strong capabilities to manipulate electromagnetic (EM) waves at frequencies ranging from microwave to visible.^[^ ^6^, ^7^, ^8^ ^]^ According to Huygens’ principle, EM waves scattered by different meta‐atoms in a metasurface can gain specific phases determined by the pre‐encoded phase profile, and thus the interferences among these scattered waves can form a new wavefront in the desired manner.^[^ ^9^, ^10^ ^]^ Many fascinating effects were demonstrated to control linearly^[^ ^6^, ^7^, ^11^, ^12^, ^13^, ^14^ ^]^ or circularly^[^ ^15^, ^16^, ^17^, ^18^, ^19^, ^20^, ^21^, ^22^, ^23^, ^24^, ^25^ ^]^ polarized EM waves based on metasurfaces with encoded phases generated by resonant mechanism or Pancharatnam–Berry (PB) mechanism. These meta‐devices are flat and ultra‐compact, being promising candidates for integration‐optics applications.

However, most metasurfaces proposed in early days exhibit only one specific functionality. To overcome this issue, metasurfaces possessing polarization‐multiplexed functionality were recently proposed.^[^ ^18^, ^26^, ^27^, ^28^, ^29^, ^30^, ^31^, ^32^, ^33^, ^34^, ^35^, ^36^, ^37^, ^46^, ^47^, ^48^ ^]^ Constructed by meta‐atoms exhibiting both resonant and PB phases, these metasurfaces can possess two distinct pre‐designed spin‐dependent phase profiles. Various multiplexed metasurfaces working at different frequencies were realized in reflection^[^ ^18^, ^30^, ^31^, ^46^ ^]^ or transmission configurations,^[^ ^27^, ^28^, ^29^, ^31^, ^32^, ^33^, ^34^, ^35^, ^36^, ^37^, ^47^, ^48^ ^]^ based on plasmonic or high‐index dielectric meta‐atoms. In viewing the performances of these multifunctional meta‐devices, however, we found that the working efficiency is a bottleneck issue, especially for transmission‐mode devices. To ensure a high working efficiency for a transmissive device, each meta‐atom should exhibit a 100% polarization conversion ratio (PCR) and a high transmission amplitude for light, as dictated by the PB mechanism.^[^ ^21^ ^]^ Meanwhile, the constitutional meta‐atoms should also possess resonant phases covering a wide range, in order to achieve the desired wavefront‐reshaping multi‐functionalities. Unfortunately, these requirements are difficult to meet simultaneously in practical designs. For example, plasmonic metasurfaces are highly lossy especially working in the transmission mode.^[^ ^23^, ^38^ ^]^ Although dielectric metasurfaces are free of absorption losses,^[^ ^10^, ^33^, ^39^, ^40^, ^41^, ^42^ ^]^ presently adopted meta‐atoms are usually of low aspect ratio (AR) and thus they are difficult to accommodate high PCR, wide transmission‐phase range, and high transmittance of THz light simultaneously.

**Figure 1:**
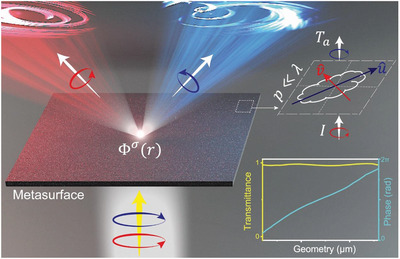
Schematics of the transmissive high‐efficiency bifunctional metasurface, constructed by meta‐atoms exhibiting both high anomalous‐mode generation efficiency *T*_a_ and wide ranges of transmission‐phase variations.

In this paper, we experimentally realize a series of dielectric metasurfaces that can achieve spin‐modulated bifunctional manipulation on circularly polarized (CP) THz waves, exhibiting working efficiencies much higher than previous attempts. As schematically shown in **Figure** **1**, the most crucial element in our study is the high‐AR (20:1) silicon pillar adopted as our building block, which, upon tuning lateral geometries, exhibits a much wider transmission‐phase variation range and a high‐PCR range, as compared to their low‐AR counterparts. As a proof of concept, we first fabricate a metasurface with only PB phases encoded and experimentally demonstrate that it can achieve photonic spin Hall effect (PSHE) with a record‐high absolute efficiency of 92%. We next realize a spin‐multiplexed metasurface with both resonant and PB phases encoded, and experimentally demonstrate that it can either anomalously refract or focus incident CP THz wave depending on the helicity of incident beam, with an absolute efficiency of up to 88%. Finally, we experimentally demonstrate spin‐multiplexed dual meta‐holograms, based on the third meta‐device designed/fabricated to exhibit two spin‐dependent phase profiles. Full wave simulations are in nice agreement with all experimental results.

## 2 Results and Discussions

### 2.1 Basic Concept

**Figure 2:**
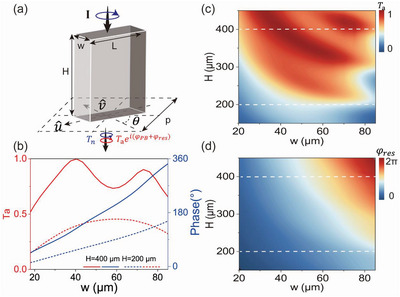
Generic phase diagrams of the silicon‐pillar meta‐atom. a) Schematics of the silicon micropillar. b) Numerically computed the anomalous‐mode generation efficiency (*T*_a_) and transmission phase of two silicon micropillars arranged periodically of different heights (*H* = 200 and 400 µm) when changing the width (*w*) of micropillars. Numerically computed c) anomalous‐mode generation efficiency *T*_a_ and d) resonance phase of periodic arrays of silicon micropillars with varying *w* and *H*. In all cases studied, *L* = 132 µm, *p* = 157 µm and the frequency is 0.65THz.

We start by introducing our strategy to design high‐efficiency spin‐multiplexed metasurfaces. Consider a silicon pillar exhibiting a rectangle cross‐section (see **Figure** **2**a) as our basic meta‐atom. The transmission and reflection properties of such a dielectric pillar can be generally described by the following Jones’ matrices: $T=\left(\begin{array}{cc} t_{uu} & 0\\ 0 & t_{vv} \end{array}\right),\;R=\left(\begin{array}{cc} r_{uu} & 0\\ 0 & r_{vv} \end{array}\right)$, where *u* and *v* denote two principle axes, respectively. Rotating the meta‐atom by an angle of *µ* with respect to the z‐axis and illuminating it by a circularly polarized (CP) light, wave scattered by such a meta‐atom generally consists of four parts, which are two spin‐conserved normal modes at the transmission and reflection sides with power efficiencies denoted as *T*_n_ and *R*_n_, and two spin‐flipped anomalous modes at the transmission and reflection sides with power efficiencies denoted as *T*_a_ and *R*_a_, respectively. According to refs. ^[^ ^21^, ^22^ ^]^, these power efficiencies take the following analytical forms:

(1) $$\begin{array}{c} T_{a}={\left|\left(t_{uu}-t_{vv}\right)\right|}^{2}\;/4,\;R_{a}={\left|\left(r_{uu}-r_{vv}\right)\right|}^{2}\;/4\\ T_{n}={\left|\left(t_{uu}+t_{vv}\right)\right|}^{2}\;/4,\;R_{n}={\left|\left(r_{uu}+r_{vv}\right)\right|}^{2}\;/4 \end{array}$$

Furthermore, it is shown in ref. [21] that two anomalous modes exhibit geometric Pancharatnam–Berry (PB) phases which can be utilized to achieve the desired wave‐control functionalities, while two normal modes do not carry such phases. Therefore, to achieve 100% efficiency manipulation on CP light at the transmission side, we need to suppress all unwanted modes and retain only the anomalous transmission mode (i.e., *R*_a_ = *R*_n_ = *T*_n_ = 0, *T*_a_ = 1). Thus, our meta‐atoms should satisfy the following criteria,

(2) $$\left|r_{\mathrm{uu}}\right|=\left|r_{\mathrm{vv}}\right|=0,\;\left|t_{\mathrm{uu}}\right|=\left|t_{\mathrm{vv}}\right|=1,\;\arg\left(t_{\mathrm{uu}}\right)-\arg\left(t_{\mathrm{vv}}\right)=\pi$$

implying that they must behave as ideal transmissive half‐wave plates without reflections. Supposing that a series of meta‐atoms satisfying the above requirements are designed, then waves scattered by them only contain the anomalous transmission modes with complex amplitudes given by

(3) $${\tilde{t}}_{a}=\left(\frac{t_{uu}-t_{vv}}{2}\right)\;e^{i\sigma 2\theta }=\sqrt{T_{a}}\;e^{i\Phi_{\mathrm{tot}}^{\sigma }}$$

where the total transmission phase is

(4) $$\Phi_{\mathrm{tot}}^{\sigma }=\sigma \cdot \;\varphi_{\mathrm{PB}}+\varphi_{\mathrm{res}}=\sigma \cdot 2\theta +\arg\left(t_{\mathrm{uu}}-t_{\mathrm{vv}}\right)$$

Obviously, now the total transmission phase of a meta‐atom contains two parts, a spin‐dependent PB phase determined by the orientation angle *θ*, and a spin‐independent phase *φ*_res_ dictated by the geometrical parameters of the silicon pillar usually related to certain resonances. Here, *σ* represents the spin states of the incident THz CP light (*σ* = + 1: left circular polarization (LCP); *σ* = − 1: right circular polarization (RCP)).

We thus establish a general strategy to realize high‐efficiency bifunctional meta‐devices working in transmission mode. Given two arbitrary pre‐designed wave‐control functionalities with phase distributions given by $\Phi_{\mathrm{tot}}^{\sigma }\left(r\right)$, we can easily retrieve the required PB and resonant phase distributions {*φ*_PB_(***r***),*φ*_res_(***r***)} for the metasurface under design, which in turn, assist us to determine the orientation angles and geometric parameters of the desired meta‐atoms that satisfying the criterion Equation (2). With all meta‐atoms determined, we then finish the design of the bifunctional meta‐device with high performance.

### 2.2 Design, Fabrication, and Characterization of the Meta‐Atoms

We now discuss how to design meta‐atoms satisfying the criteria presented in the last sub‐section. First of all, Equation (2) requires that the designed meta‐atoms should possess largest possible values of *T*_a_, which not only ensures a high working efficiency of the fabricated device, but also eliminates all unwanted modes to improve the performance of the wave‐control functionality. Meanwhile, Equation (4) suggests that the meta‐atoms should also exhibit transmission phases varying in a sufficiently large range, which is crucial to achieving the pre‐designed wave‐control bifunctionalities. However, these two requirements are extremely difficult to meet simultaneously in dielectric pillars with low aspect ratio (AR). To clearly illustrate this point, we employ full wave simulations to compute the transmission properties of silicon pillars with different heights *H* and widths *w* (with length *L* = 132 µm fixed), which are arranged in a square lattice with periodicity *p* = 157 µm. Figure 2c,d depicts, how the calculated value of *T*_a_ and *φ*_res_ of the dielectric pillar vary as functions of *H* and *w*, at the working frequency 0.65 THz. Clearly, for micropillars with relatively low AR (say, *H* = 200 µm), varying the parameter *w* within (18 −85µm) can change the transmission phase *φ*_res_ inside a range of 140°, but with working efficiencies *T*_a_ restricted in a low‐value range (0.15–0.45). In sharp contrast, as the AR of our micropillar increases, the variation range of *φ*_res_ with high‐*T*_a_ is significantly enlarged, which offers much wider designing freedoms. Taking *H* = 400 µm as an example, we find that the variation range of *φ*_res_ reaches ≈180° with *T*_a_ > 0.75, as *w* is varied from 20 to 65 µm. The inherent physics can be explained by the following arguments. Considering the micropillar as a dielectric waveguide, transmission phase of THz light passing through it is roughly proportional to the height *H* of the waveguide, subject to a cut‐off wavelength dictated by the lateral size of the pillar. Therefore, both the individual values of *φ*_*uu*_, *φ*_*vv*_ and their difference Δ*φ* = *φ*_*uu*_ − *φ*_*vv*_, which are intrinsically related to *φ*_res_ and *T*_a_, respectively, are roughly linearly dependent on *H*. Adjusting lateral dimension *w* of the pillar can tune these phases through modifying the cut‐off wavelengths, but it is easy to expect that the variation slopes of these phases upon tuning *w* must be larger in larger‐*H* cases, which explains why we have a wider tuning range of *φ*_res_ with a larger *T*_a_ in a higher‐AR case.

**Figure 3:**
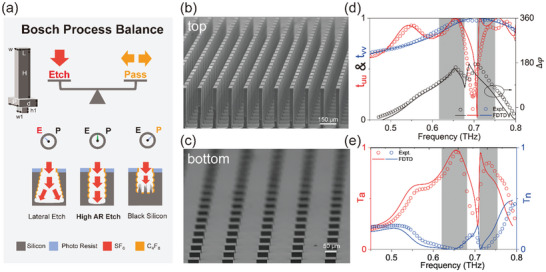
Design, fabrication, and characterization of a realistic meta‐atom. a) To fabricate our high‐quality tri‐layer meta‐atoms with high aspect ratios (inset), a Bosch process with balanced etch phase (Etch) and passivation phase (Pass) is adopted. Lateral etch occurs as Etch > Pass, while black silicon is formed as Pass > Etch. High‐AR etch can only be achieved under the balanced condition. b,c) The top‐view and bottom‐view SEM pictures of the fabricated metasurface consisting of a periodic array of meta‐atoms with structural parameters *H* = 400 µm, *w* = 33 µm, *L* = 132 µm, *p* = 157 µm, *d* = 62 µm, *h*1 = 38 µm, *w*1 = 50 µm. d) Spectra of transmission amplitudes (*t_uu_* and t_*vv*_) and phase difference Δ*φ*( = *φ*_*uu*_ − *φ*_*vv*_) of the designed/fabricated metasurface, obtained by simulations (lines) and measurements (circles). e). Spectra of anomalous‐mode and normal‐mode generation efficiency (*T*_a_ and *T*_n_) of the designed meta‐atom, retrieved from the transmission obtained by simulations (solid lines) and measurements (open stars).

Having understood the basic requirements of meta‐atoms and their governing physics, we next design and fabricate such silicon micropillars with very high AR. Since in practice our micro‐pillars must be deposited on a substrate, we add carefully designed silicon micro‐resonators to another side of the substrate, serving as an anti‐reflection film to reduce the reflections due to the substrate. Therefore, our meta‐atom is designed to consist of three layers (see the inset to **Figure** **3**a) all made of high‐resistance silicon (with *n*_si_ = 3.45), including a 400 µm − high micro‐pillar with a rectangle cross‐section, a 62 µm – thick continuous substrate, and a 38 µm − high micro‐resonator exhibiting a square cross‐section with dimension optimized to yield the highest transmission for the whole meta‐atom at the target working frequency (see detailed discussions in Section S(C), Supporting Information).

We fabricate out a metasurface consisting of a periodic array of the designed meta‐atoms as depicted in Figure 3a, using photolithography (Karl Suss MA6 Mask Aligner) and deep reactive ion etch (Bosch process) on a 500 µm − thick silicon wafer following the process schematically shown in Figure S14, Supporting Information. The desired patterns are transferred onto the wafer through the Bosch process, a time multiplexed deep etching technique^[^ ^43^, ^44^ ^]^ consisting of both etch phase and passivation phase (see Figure S14, Supporting Information), respectively. A crucial step is that we carefully balance the two phases in order to fabricate out the high‐AR microstructures with the best quality (see Figure 3a).^[^ ^45^ ^]^ If the etch phase suppresses the passivation one, fabricated pillars can exhibit a trapezoid shape and thus be very fragile. Meanwhile, if the passivation phase exceeds the etch one, black silicon (micro grass) can be formed in between two pillars which may deteriorate the sample quality.^[^ ^38^, ^44^ ^]^ After carefully balancing these two phases, we finally fabricate out the desired metasurface consisting of high‐AR (about 20:1) microstructures with smooth surfaces, straight sidewalls, and nice alignment. Figure 3c,d depicts the top‐view and bottom‐view scanning electron microscopy (SEM) pictures of the fabricated sample, respectively.

We employ THz time‐domain spectroscopy (TDS) to characterize the transmission properties of the fabricated metasurface. Red and blue circles in Figure 3d depict the measured spectra of transmission amplitude of the sample for two incident polarizations, while black circles represent the measured spectrum of transmission‐phase difference between two polarizations. All measured results are in excellent agreement with finite‐difference time‐domain (FDTD) simulations on realistic structures (lines). We find clearly that there are two frequency windows ([0.63–0.66 THz] and [0.72–0.76 THz]) where the conditions |*t_uu_* |, |*t_vv_* | ≈ 1 and Δ*φ* ≈ 180 are approximately satisfied, meeting the requirement of Equation (2). The intriguing low‐performance regime around 0.69 THz is caused by a Fabry‐Perot (FP) resonance inside the anti‐reflection layer, which can be remedied by the top‐micropillar rotates at an angle relative to the bottom structure and designing the shapes of micro‐resonators (See Figure S3(d), Supporting Information). The working bandwidth of the present meta‐device is limited by multiple scattering of light inside the anti‐reflection structure, but can be further increased via optimizing the anti‐reflection structure to shift its FP mode away (see Figure S4, Supporting Information). Substituting the measured transmission and reflection coefficients into Equation (1), we obtain the spectra of power efficiencies for four different modes, and depict the measured spectrum of *T*_a_ in Figure 3e. We find from Figure 3e that *T*_a_ reaches 0.99 at 0.65 THz, implying that our meta‐atom exhibits a nearly 100% efficiency at this frequency, with all unwanted modes suppressed. We emphasize that *T*_a_ represents the absolute efficiency of such a PB meta‐atom, which measures the ratio between power carried in the anomalous mode and that of the incident one.

### 2.3 High‐Efficiency Photonic Spin‐Hall Effect

**Figure 4:**
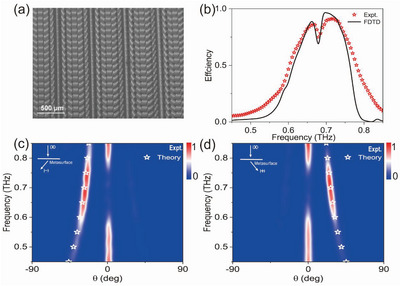
Experimental characterizations of the high‐efficiency PSHE meta‐device. a) Top‐view SEM picture of the fabricated sample consisting of meta‐atoms designed in Figure 3 exhibiting spatially varying orientation angles. b) Absolute working efficiency of the device versus frequency, retrieved from experimentally measured and simulated scattering patterns using a power‐integration method. Color maps are normalized scattering‐power distributions measured with c) an LCP detector or d) an RCP detector at the transmission side, as the metasurface is shined by linearly polarized THz waves under normal incidence. Open stars represent the positions predicted by the generalized Snell's law.

As a benchmark test, we employ the meta‐atom designed in the last section to realize a dielectric PB metasurface and experimentally demonstrate that it can achieve PSHE with a record‐high efficiency. As shown in **Figure** **4**a, the fabricated metasurface consists of identical high‐AR micro‐pillar meta‐atoms with orientation angles changing linearly in space with inter‐meta‐atom difference Δ*ϕ* = *π*/6. According to the PB mechanism, we immediately understand that such a metasurface exhibits the following phase distribution

(5) $$\Phi^{\sigma }\;\left(x\right)=\varphi_{0}\;+\sigma \xi \cdot x$$

with ξ=2·Δϕ/p being the phase gradient. Generalized Snell's law^[^ ^6^, ^7^ ^]^ thus predicts that this metasurface can refract a normally incident CP THz beam with spin *σ* to an off‐normal direction $\theta_{t}^{\sigma }=\sin^{-1}\;\left(\sigma \xi /k_{0}\right)$ where *k*_0_ is the free‐space wave vector. Therefore, under the illumination of a linearly polarized (LP) THz beam, we expect our metasurface to bend the LCP and RCP components inside the LP beam to opposite directions at the transmission side, giving rise to the so‐called PSHE.

We experimentally demonstrate the predicted effect using a THz angle‐resolved TDS. In our experiment, shining an LP THz beam normally onto the sample from the bottom side, we measure the *x*‐ and *y*‐polarized components of scattered electric fields (including both amplitudes and phases) at different diffraction angles with a detector moving on a motorized rotation stage. Transforming the measured data to circular‐polarization electric‐field components $E^{\sigma }=\;\left(E_{x}-i\sigma E_{y}\right)/\sqrt{2}$, we then obtain the normalized scattered power distributions for transmitted RCP and LCP lights, and depict them in Figure 4c,d. Here, all data are normalized against the reference signals obtained by detecting the CP light transmitted through an aperture with the same size as the metasurface, under the same illuminations. Clearly, RCP and LCP components of transmitted THz light are bent to off‐normal directions agreeing well with the predictions of the generalized Snell's law, while the normally transmitted waves are deeply suppressed within the frequency band of [0.63–0.75 THz], already implying the high efficiency of our device.

We have quantitatively evaluated the working efficiency of the fabricated metasurface. Integrating the power of anomalous‐transmission light within the angle range around $\theta_{t}^{\sigma }$ to obtain a value of *P*_a_, and performing the same procedure to obtain a reference value *P*_in_ with the metasurface replaced by an aperture of the same size, the ratio between them *P*_a_/*P*_air_ is thus the absolute efficiency of our device. Open stars in Figure 4b depict the retrieved absolute working efficiency of our device with varying frequency, which are in nice agreement with FDTD simulation results (line in Figure 4b) on realistic structures (see more details in Section S(D), Supporting Information). We note that our device exhibits a maximum efficiency of 92% at 0.7 THz (94% in simulations), which is a record‐high value in literature. Overall, the fabricated PB metasurface exhibits a similar frequency‐dependent efficiency as the *T*_a_ spectrum of the meta‐atom (see Figure 4b). Interestingly, we note that the low‐performance dip around 0.69 THz in the *T*_a_ spectrum of the meta‐atom is largely alleviated in the PB sample. This is probably caused by a different arrangement of meta‐atoms in the PB sample as compared to the periodic sample, which partially destroys the FP resonance in the metasurface containing a periodic array of meta‐atoms studied in Figure 3.

### 2.4 Bifunctional Meta‐Devices for Wavefront Reshaping and Holography

**Figure 5:**
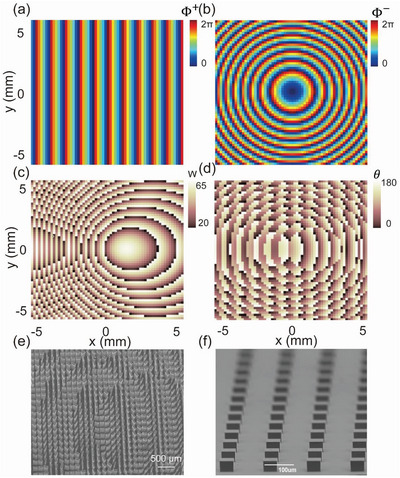
Design and fabrication of the high‐efficiency bifunctional meta‐device for light bending and focusing. Desired phase distributions a) Φ^+^(r) and b) Φ^−^(r) exhibited by the metasurface as shined by incident light with LCP and RCP, respectively. At the working frequency 0.65 THz, retrieved geometry‐size distribution c) *w*(r) and rotation‐angle distribution d) *θ*(*r*) of the meta‐atoms forming the meta‐device. e) Top‐view and f) bottom‐view SEM pictures of the fabricated sample according to the *w*(r) and *θ*(*r*) distributions as shown in (c) and (d).

Encouraged by the excellent performance of the PB metasurface, we continue to design spin‐delinked bifunctional meta‐devices that can exhibit two distinct phase profiles for excitation of THz CP light with a different spin. Two different functions can be achieved in a single metasurface as illuminated by the input light of different chirality. The first device that we design is a metasurface exhibiting the following spin‐dependent phase profiles

(6) $$\left\{\begin{array}{c} {\;\Phi }^{-}=\varphi_{0}-k_{0}\cdot x\sin\theta_{r}\\ {\;\Phi }^{+}=-k_{0}\cdot \left(\sqrt{x^{2}+y^{2}+F^{2}}-F\right) \end{array}\right.$$

at the working frequency 0.65THz with *F* = 2 mm and $\theta_{r}=30^{\circ }$. Obviously, such a meta‐device is designed to work as a light bender for incident THz beam with LCP and as a flat lens for the RCP excitation, dictated by Φ^*σ*^(*x*,*y*) distributions as shown in **Figure** **5**a,b. We can then easily retrieve the two phase profiles Φ^PB^(*x*,*y*) and Φ^Res^(*x*,*y*) from Φ^*σ*^(*x*,*y*) according to Equation (6). The Φ^PB^(*x*,*y*) distribution helps us obtain the rotation angle *θ*(*x*, *y*) distribution of our meta‐atoms, as shown in Figure 5c. Meanwhile, according to the phase diagram Figure S6, Supporting Information, that relates resonance phase Φ^Res^ with structural parameter w of the meta‐atom, we can retrieve the w(*x*, *y*) distribution (Figure 5d) from the Φ^Res^(*x*,*y*) profile to determine the structural parameter w of all our meta‐atoms. We note that only the parameter w is varied in designing our meta‐atoms, with other parameters kept unchanged. With the other two parameters (*H* and *L*) fully relaxed, one can certainly get a design with a higher efficiency, but unfortunately, the challenge in fabricating such a structure is significantly increased. The present design strategy makes fabrications easy, yet still keeps all meta‐atoms exhibiting reasonably high efficiency (see Figure S6, Supporting Information). Employing the manufactural technology described in previous sub‐section, we successfully fabricate the bifunctional meta‐device based on the two‐parameter distributions as shown in Figure 5c,d. Figure 5e,f depict the bottom‐ and top‐view SEM pictures of the fabricated sample, respectively.

**Figure 6:**
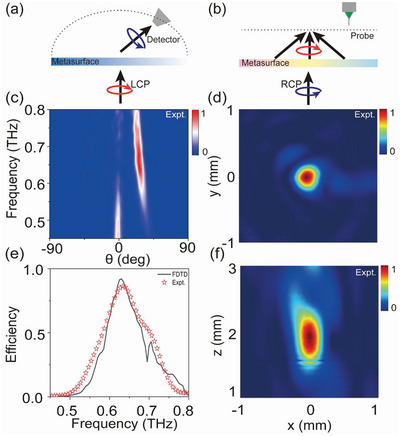
Experimentally characterization of the high‐efficiency bifunctional device. Schematics of the two functionalities of our meta‐device as shined by a) LCP and b) RCP light. c) Measured normalized scattering‐power distributions at different frequencies as the meta‐device is shined by LCP light. e) Absolute working efficiency of the device as a function of frequency, retrieved from experimentally measured (open stars) and simulated (solid line) scattering patterns using a power‐integration method. Measured field distributions on the d) xy‐plane with z = 2 mm and the f) yz‐plane with x = 0 mm plane as the device is illuminated by RCP light at 0.65 THz.

We perform both far‐field and near‐field measurements to characterize the functionalities of the fabricated meta‐device. Shining the sample by normally incident THz light with LCP, we adopt a THz angle‐resolved TDS system to characterize the light‐bending functionality of our device, as schematically shown in **Figure** **6**a. Using similar characterization techniques as in Figure 4, we measure the normalized scattering pattern of the total transmitted wave at different frequencies, and depict the results in Figure 6c. At general frequencies, there are two scattering beams on the transmission side, corresponding to a normal and an anomalous mode taking LCP and RCP polarizations, respectively. Within the frequency band [0.60–0.66 THz], however, we find that the impinging LCP light has been efficiently deflected to a single anomalous mode traveling to the off‐normal direction dictated by the generalized Snell's law, with the normal mode completely suppressed. This already implies a high working efficiency of the fabricated device. Further, we employ the power‐integration technique to quantitatively evaluate the absolute working efficiencies of our device at different frequencies, retrieved from both measured and simulated scattering patterns. Figure 6e shows that experimental results are generally in good agreement with simulation ones. In particular, the maximum experimental efficiency of our device can reach 88% at 0.63THz, while the simulation one is 91%.

We next characterize the second wave‐manipulation functionality of our device under the RCP illumination, utilizing a THz near‐field scanning system as schematically shown in Figure 6b. Here, normally shining the device by an RCP beam converted from a linearly polarized THz beam through a quarter‐wave plate, we adopt a near‐field detector to measure the electric‐field distributions on the *xy*‐plane with z = 2 mm and the *yz*‐plane with x = 0 mm, respectively. Figure 6d–f depicts the measured distributions of |*E_x_* | on the above‐mentioned two planes, at the working frequency of 0.65THz. Clearly, the incident RCP wave has been well focused to the center point on the z = 2 mm plane after passing through our meta‐device, in nice agreement with theoretical predictions (see Equation (6)). Simulation results are in good agreement with measured ones (see Figure S8, Supporting Information), which undoubtedly verify the good performances of our device. More experimental results at different frequencies are shown in Figure S10, Supporting Information.

**Figure 7:**
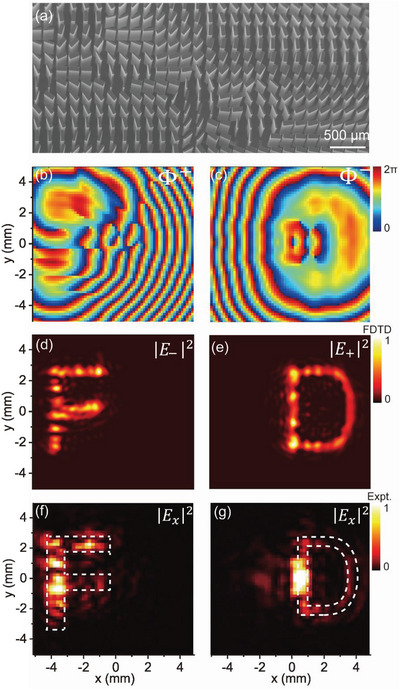
Experimental characterization of the high‐efficiency bifunctional meta‐hologram. a) Top‐view SEM picture of the fabricated sample. Desired phase distributions b) Φ^+^(r) and c) Φ^−^(r) of the metasurface under design. FDTD simulated images on the hologram plane as the metasurface is shined by THz waves with d) LCP and e) RCP, respectively. Experimentally measured energy of x‐component of E‐field (|*E_x_* |^2^) on the hologram plane as the metasurface is shined by THz waves with f) LCP and g) RCP, respectively. Here, the working frequency is 0.65THz.

Our design scheme is so general that it can be employed to realize bifunctional meta‐devices with more complex wave‐manipulation capabilities. As an illustration, we now experimentally demonstrate a high‐efficiency THz meta‐device that can achieve distinct hologram images, under the excitations of CP THz beams with different spins. Assuming that two target holographic images are letters “F” and “D” appearing on the plane z = 4.5 mm as the metasuface is shined by LCP and RCP light, respectively, we retrieve the required phase distributions Φ^+^(*x*,*y*) and Φ^−^(*x*,*y*) (see **Figure** **7**b,c) possessed by the metasurface using the classical Gerchberg–Saxton algorithm, setting the working frequency as 0.65 THz. From the Φ^*σ*^(*x*,*y*) distributions we then obtain Φ^Res^(*x*,*y*) and Φ^PB^(*x*,*y*), which finally assist us to determine the geometrical‐parameter distribution w(*x*, *y*) and the rotation‐angle distribution *θ*(*x*, *y*) (see Figure S11, Supporting Information), required for the sample design. In designing the realistic metasurface sample, we discretize the whole space into pixels with deep‐subwavelength size of 157 µm × 157 µm, each containing meta‐atoms with structural parameters and orientational angles dictated by the values of w(*x*, *y*) and *θ*(*x*, *y*) on the center of the pixel. We fabricate out the meta‐device according to the design, with its top‐view SEM picture shown in Figure 7a. We next use the same near‐field characterization technique as in Figure 6d,f to measure the holographic images created on the image plane, as our metasurface is shined by normally incident CP THz light with a different spin. We find from Figure 7f,g that a letter “F” (or “D”) indeed appears on the image plane, as the metasurface is shined by THz light with LCP (or RCP). We also employ FDTD simulations to directly compute the holographic images generated on the image planes, as the realistic meta‐device is shined by LCP and RCP THz beams, respectively. Simulated images depicted in Figure 7d,e are in nice agreement with the measured ones. We note that the measured holographic images are not as good as the simulated ones. The main reason is that our terahertz near‐field scanning system can only detect a single linear polarization (e.g., *E_x_*) while the designed holographic images carry circular polarizations (see more discussions in Figure S12, Supporting Information). Also, the detecting resolution and the imperfections of fabricated samples can also degrade the qualities of the measured images.

## 3 Conclusion

In this paper, we propose a systematic scheme to design transmissive dielectric metasurfaces for achieving high‐efficiency and spin‐multiplexed wavefront controls on CP THz light, and experimentally realize a set of bifunctional meta‐devices employing our newly developed technique of fabricating high‐AR (20:1) silicon micropillars. As a benchmark test, we first fabricate a dielectric PB metasurface and experimentally demonstrate that it can achieve PSHE in transmission mode, exhibiting a record‐high absolute efficiency (experiment: 92%, simulation: 94%). We next design/fabricate a bifunctional meta‐device encoded with two spin‐dependent phase profiles, and experimentally demonstrate that it can realize anomalous refraction (experimental efficiency: 88%) and point focusing for incident THz CP light with a different spin. Finally, we experimentally demonstrate spin‐sensitive dual holographic images generated by the third meta‐device that we design/fabricate, under the illuminations of LCP and RCP THz light, respectively. Our results can stimulate the realizations of many high‐efficiency, ultra‐compact, and multifunctional transmissive THz devices, being highly favorable for future on‐chip photonics applications.

## Conflict of Interest

The authors declare no conflict of interest.

## Supporting information

Supporting Information

## Data Availability

The data that support the findings of this study are available from the corresponding author upon reasonable request.
