# Supplementary material for: Bifunctional Manipulation of Terahertz Waves with High‐Efficiency Transmissive Dielectric Metasurfaces
Source: Adv Sci (Weinh). 2022 Dec 9;10(4):2205499. doi: 10.1002/advs.202205499 (PMC9896063; doi:10.1002/advs.202205499)
Supplement: Supplementary file 1 — Supporting Information [file ADVS-10-2205499-s001.pdf]

ADVANCED SCIENCE
Open Access

Supporting Information

for *Adv. Sci.*, DOI 10.1002/advs.202205499

Bifunctional Manipulation of Terahertz Waves with High-Efficiency Transmissive Dielectric Metasurfaces

Zhuo Wang, Yao Yao, Weikang Pan, Haoyang Zhou, Yizhen Chen, Jing Lin, Jiaming Hao, Shiyi Xiao, Qiong He, Shulin Sun and Lei Zhou**

Bifunctional manipulation of terahertz waves with high-efficiency transmissive dielectric metasurfaces

Zhuo Wang[1 †], Yao Yao[1 †], Weikang Pan[2 †], Haoyang Zhou[1], Yizhen Chen[2], Jing Lin[1], Jiaming Hao[3], Shiyi Xiao[4], Qiong He[1], Shulin Sun[2,5], Lei Zhou[1*]*

[1]State Key Laboratory of Surface Physics and Key Laboratory of Micro and Nano Photonic Structures (Ministry of Education), Fudan University, Shanghai 200433, China.

[2]Shanghai Engineering Research Centre of Ultra Precision Optical Manufacturing, Department of Optical Science and Engineering, School of Information Science and Technology, Fudan University, Shanghai 200433, China.

[3]Institute of optoelectronics, Fudan University, Shanghai 200433, China.

[4]Shanghai Institute for Advanced Communication and Data Science, Shanghai University, Shanghai 200444, China

[5] Yiwu Research Institute of Fudan University, Chengbei Road, Yiwu City, 322000 Zhejiang, China

E-mail: sls@fudan.edu.cn, phzhou@fudan.edu.cn

[†] These authors contributed equally to this work.

Keywords: Spin-multiplexed metasurface, Dielectric, Circular polarization, Wavefront controls, Transmission configuration

A: Transmission amplitude and phase difference diagrams of silicon pillar with different heights and widths

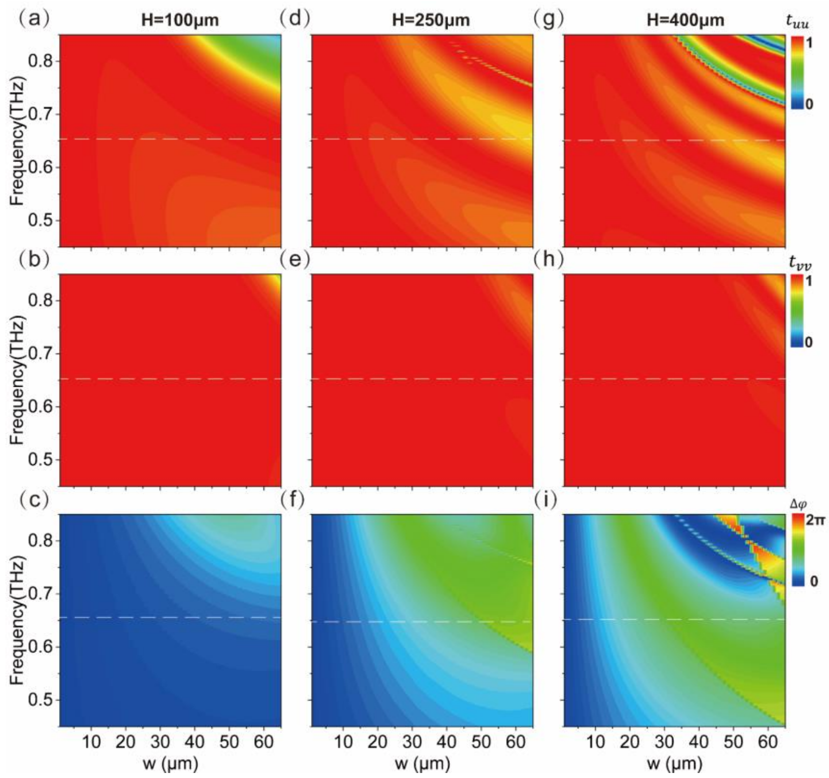

Figure S1. Transmission amplitude t_{uu}(**a**, **d**, **g**), t_{vv}(**b**, **e**, **h**) and phase difference $\Delta\varphi = \varphi_{uu} - \varphi_{vv}$ (**c**, **f**, **i**) of dielectric pillars with three different heights ($H = 100\ \mu m$ (**a-c**), $250\mu m$ (**d-f**), $400\mu m$ (**g-i**)). The other parameters are fixed: $L = 132\ \mu m, w = 33\ \mu m, p = 157\ \mu m$).

B: E field distributions inside dielectric pillars with different widths and fixed heights ($H = 400\mu m$)

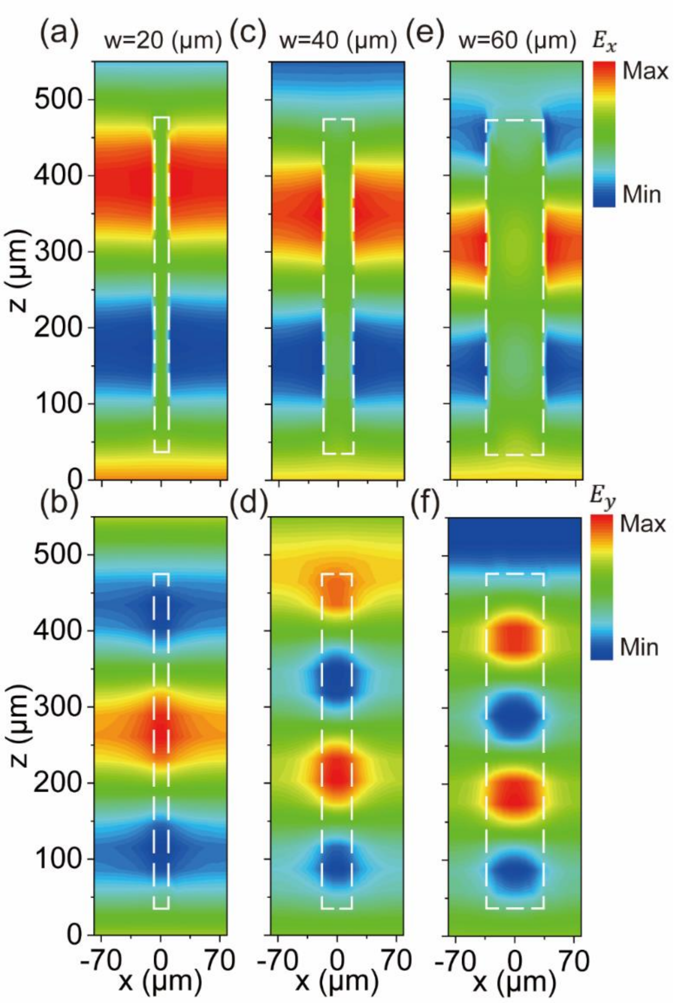

Figure S2. Real parts of E_x (**a**, **c**, **e**) and E_y (**b, d, f**) distributions of dielectric pillars with different widths $w = 20\ \mu m, 40\ \mu m, 60\ \mu m$. The other parameters are listed as follows: $H = 400\ \mu m, L = 132\ \mu m, p = 157\ \mu m$.

C: Comparison of optical properties of PB atoms with or without substrate

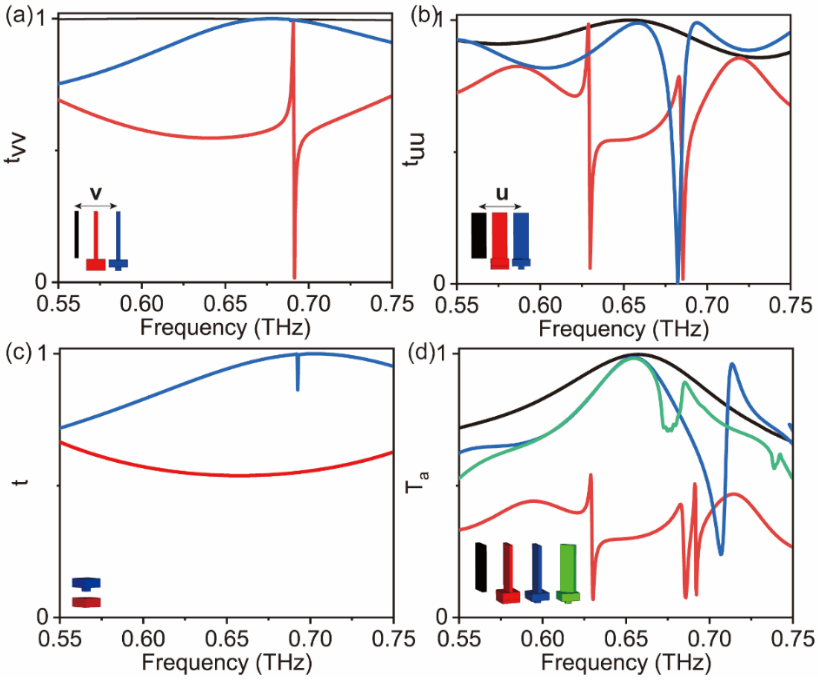

Figure S3. Transmission amplitude t_{vv} (**a**), t_{uu} (**b**), t (**c**) and T_a of different meta-atoms (see insets), including freestanding pillar (black), pillar with a homogeneous substrate (red), pillar with an anti-reflection layer (blue). (**c**) represents the transmission amplitude of the homogeneous substrate (red) and the anti-reflection layer (blue). In (**d**), the green line represents an averaged T_a results that considers the realistic structural imperfections. The structure parameters of the designed meta-atom (blue) are $H = 400\ \mu m,\ w = 33\ \mu m, L = 132\ \mu m, p = 157\ \mu m, d = 62\ \mu m, h1 = 38\ \mu m, w1 = 50\ \mu m$. All meta-atoms have the same length (L), width (w) and height (H). The thickness of homogeneous substrate (red) is the same as the total thickness of anti-reflection layer (blue).

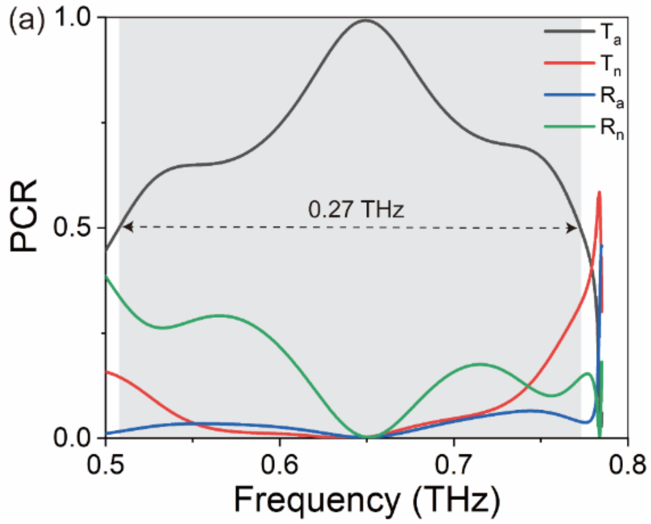

Figure S4. (a) The efficiency of normal reflection\transmission (R_n \ T_n) and abnormal reflection\transmission (R_a\$T_a$) modes of an optimized broadband PB meta-atom ($H = 400\ \mu m, w = 33\ \mu m, L = 120\ \mu m, p = 130\ \mu m, d = 62\ \mu m, h1 = 38\ \mu m, w1 = 50\ \mu m$).

D: Characterization of high-efficiency PSHE devices based on full-wave simulation

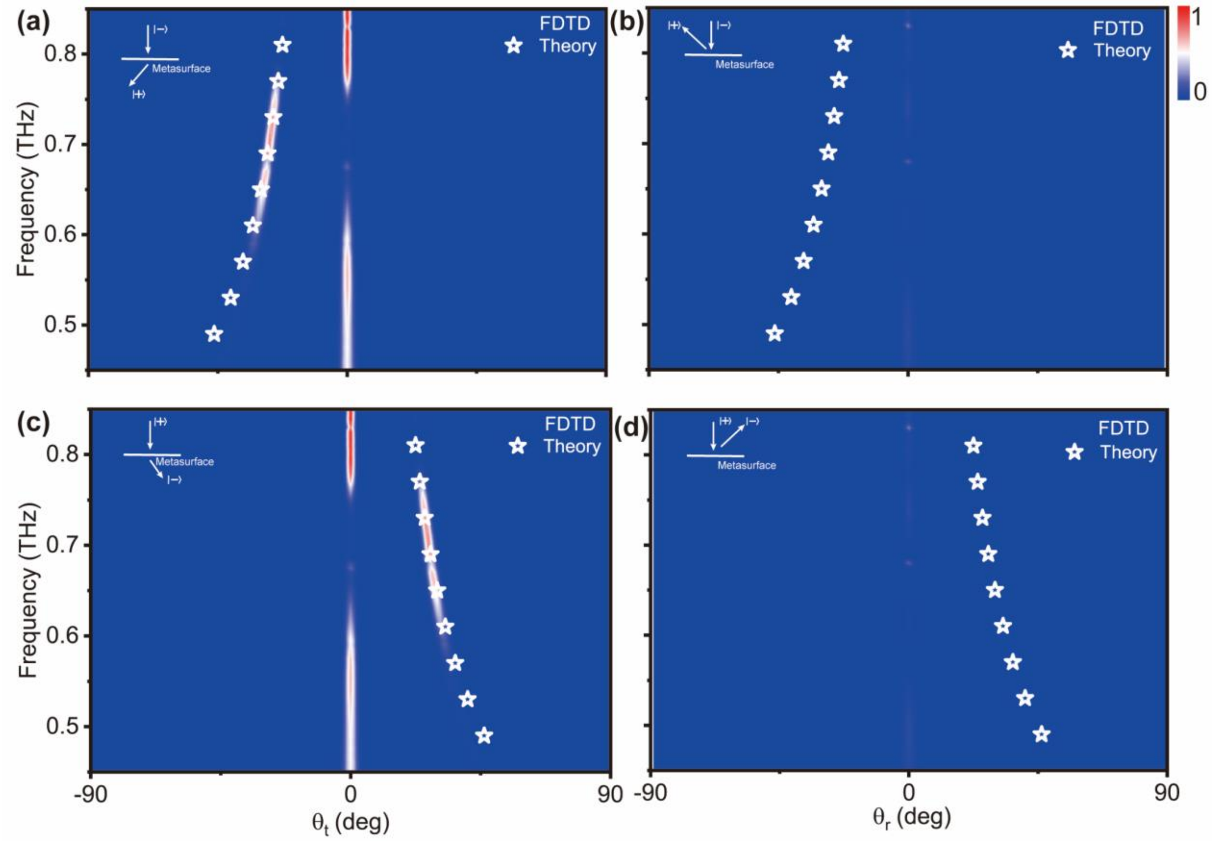

Figure S5. Normalized scattered electric field intensities carrying opposite circular polarization at the transmission (**a**, **c**) or reflection (**b**, **d**) side under the illumination of RCP (**a**, **b**) or LCP (**c**, **d**) light. Open stars represent the positions predicted by the generalized Snell's law.

E: Characterization of high-efficiency bifunctional meta-devices

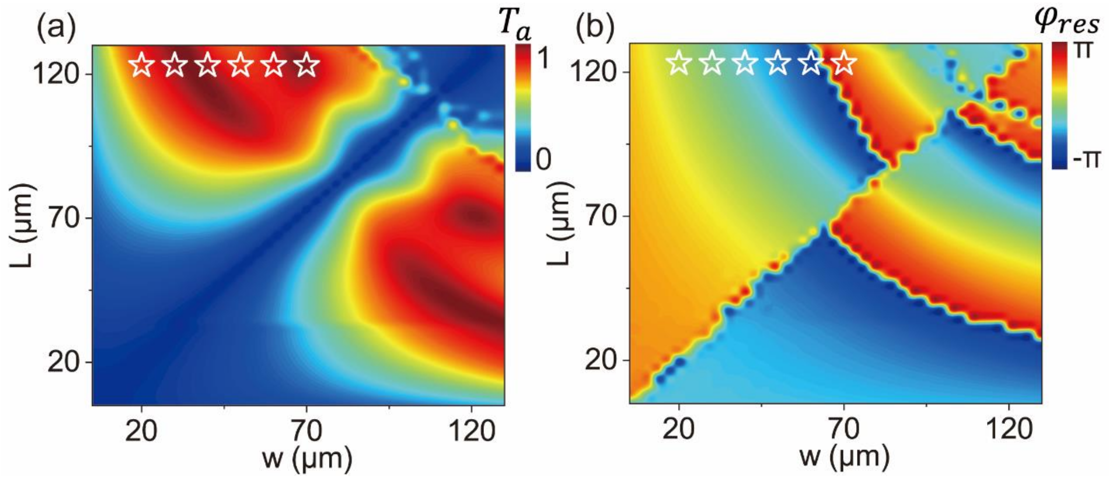

Figure S6. T_a and resonance phase of PB meta-atoms as functions of geometric parameter w and L. The adopted high-performance meta-atoms are selected from the area denoted by white stars. The other parameters are fixed: $H = 400\ \mu m, p = 157\ \mu m, d = 62\ \mu m, h1 = 38\ \mu m, w1 = 50\ \mu m$.

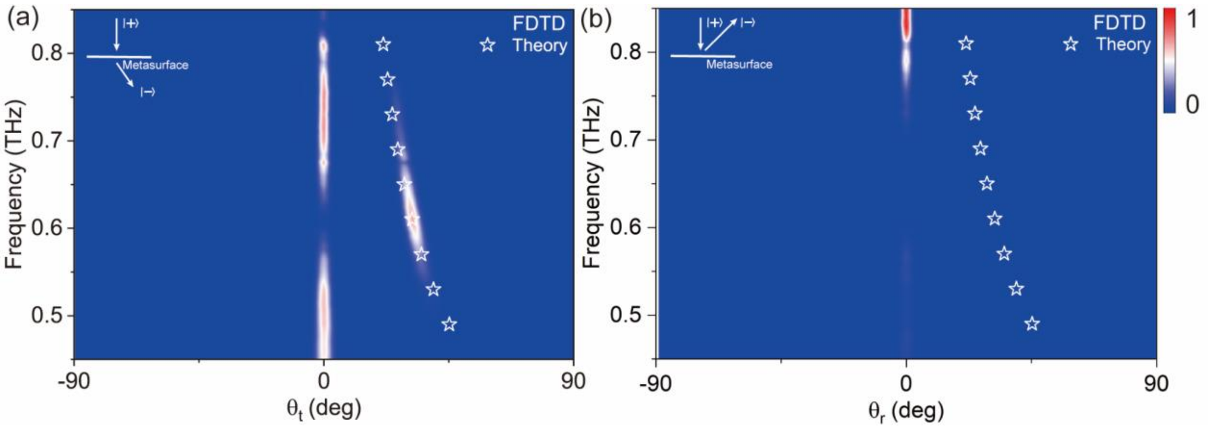

Figure S7. Normalized scattered electric field intensities carrying opposite circular polarization at the transmission (**a**) or reflection (**b**) side of the bifunctional metasurface shown in Fig. 5 under the illumination of LCP light. Open stars represent the positions predicted by the generalized Snell's law.

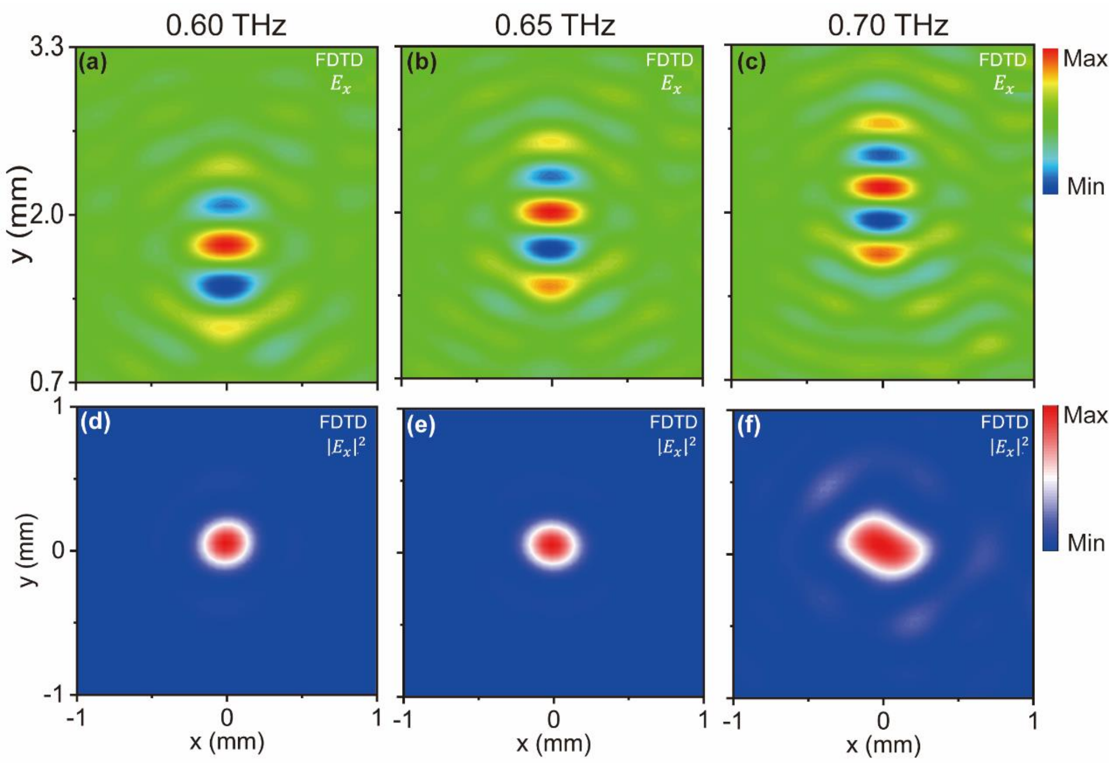

Figure S8. Numerical characterization of focusing effect of the bifunctional metasurface shown in Fig. 5 under the illumination of RCP light. Electric field distribution (**a-c**: E_x and **d-f**: $|E_x|^2$) on the plane of z=2mm (**a-c**) and x=0mm (**d-f**) obtained at different frequencies.

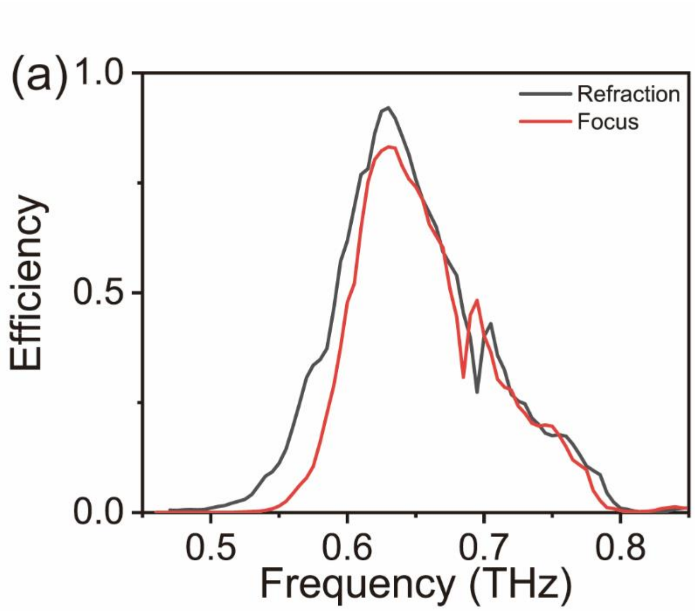

Figure S9. The calculated absolute efficiency of the bifunctional metasurface shown in Fig. 5. The red and black line represent the efficiency of refraction and focusing under the illumination of LCP/RCP light, respectively.

.

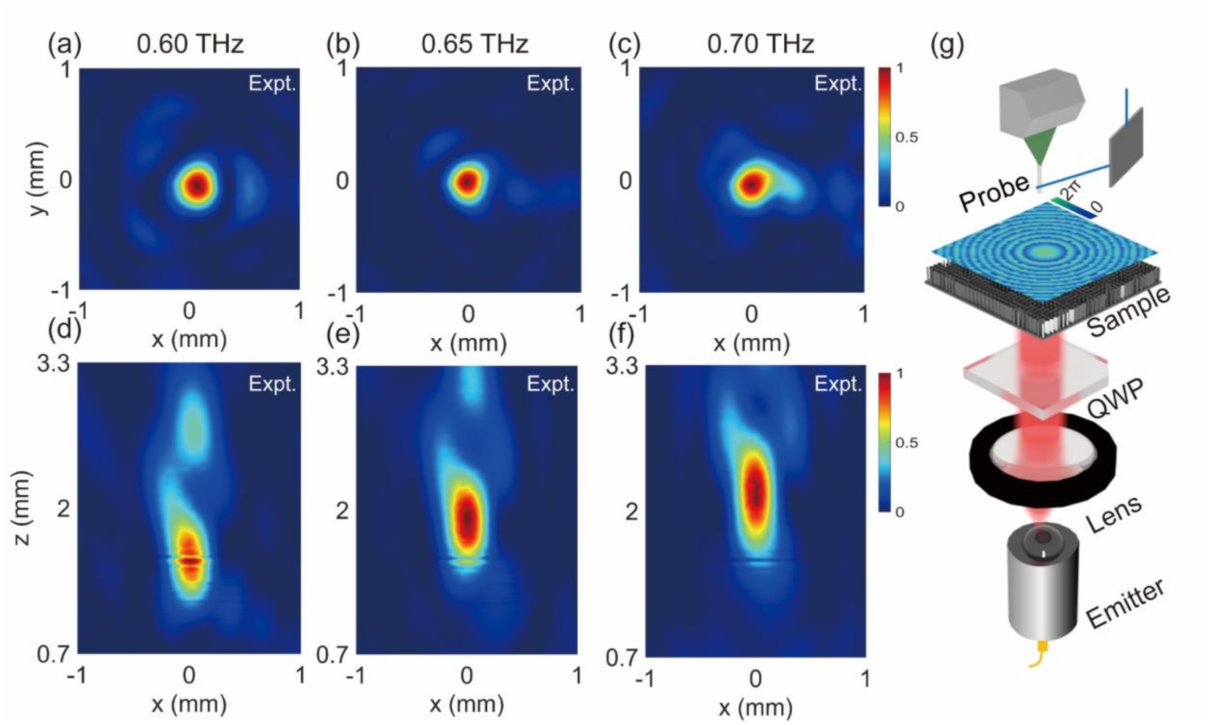

Figure S10. Experimentally measured field intensity on the z=2 mm (**a-c**) and x=0 mm (**b-d**) plane of the bifunctional metasurface shown in Fig. 5 under the illumination of RCP light at 0.60THz (**a**, **d**), 0.65 THz (**b**, **e**), 0.70 THz (**c**, **f**), respectively. **(g)** Schematic of the experimental setup of the THz near-field probe scanning system.

F: Geometry size (w) and rotation angle (θ) distributions of meta-hologram

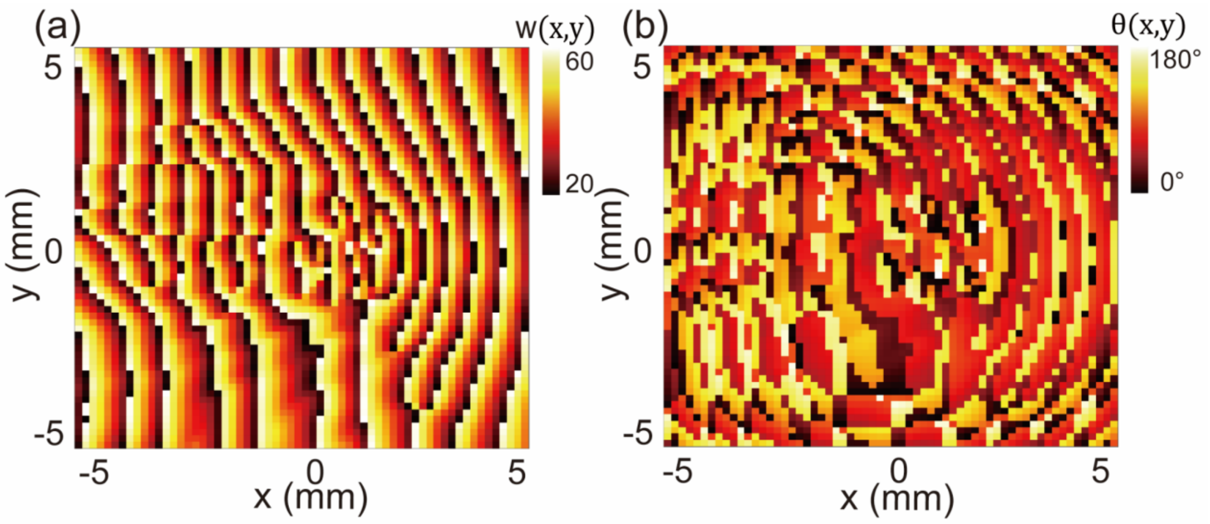

Figure S11. (a) Geometry size $w(x, y)$ and **(b)** orientation angle $\theta(x, y)$ distributions of the meta-hologram shown in Fig. 7.

G: Holographic Imaging in Different Polarization States

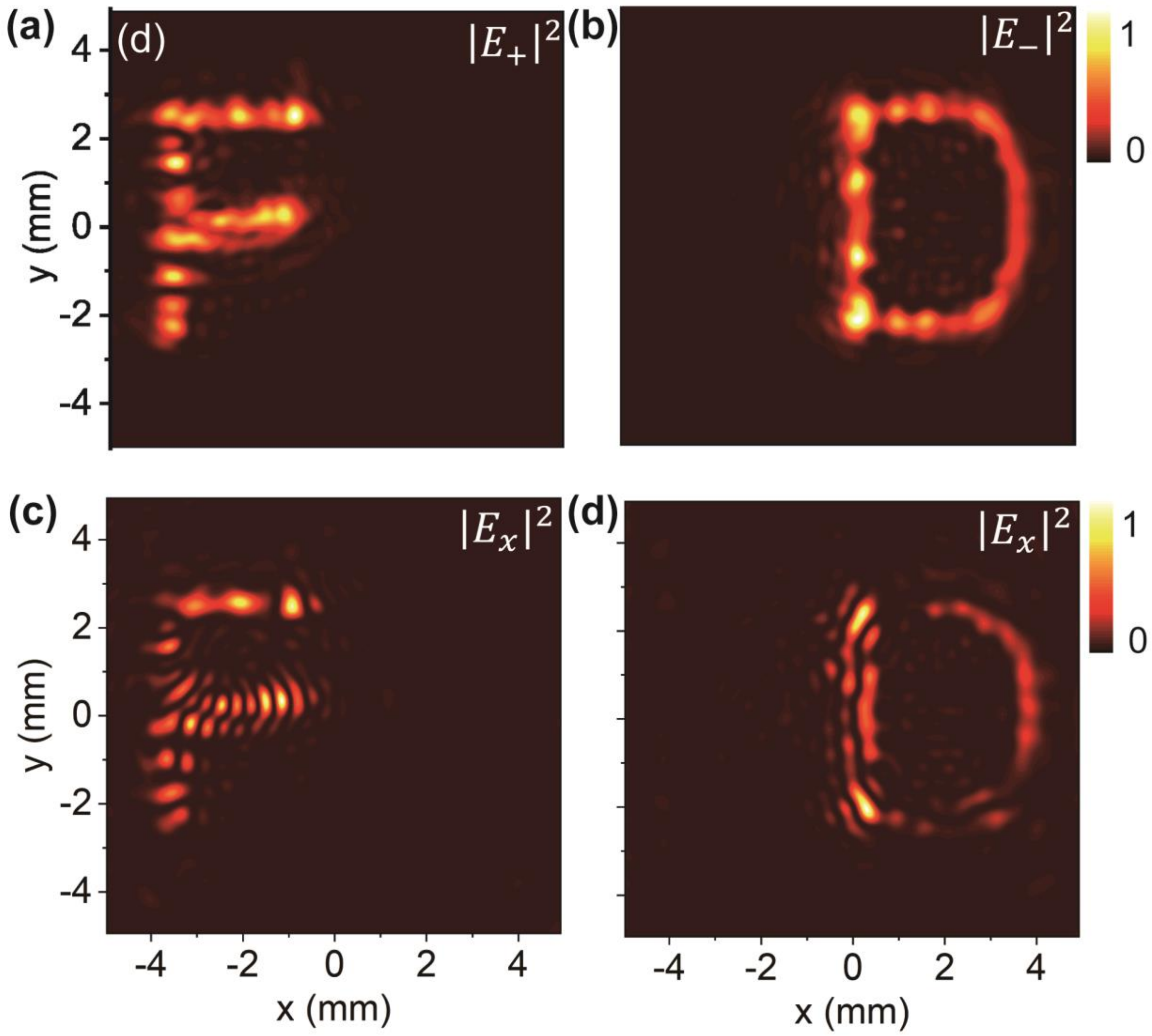

Figure S12. The calculated **(a, b)** CP polarized and **(c, d)** x-polarized electric filed intensity of the holographic images when bifunctional meta-device is illuminated by LCP/RCP THz light.

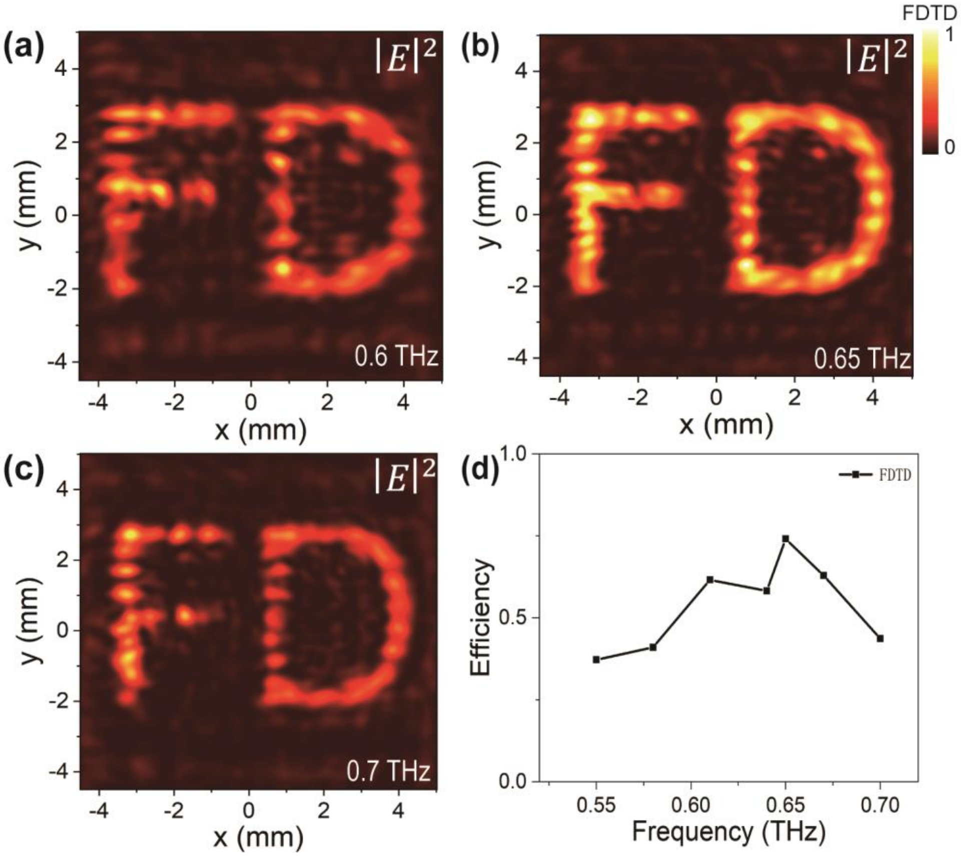

Figure S13. **(a-c)** Simulation results of holographic images generated by the meta-device illuminated by the linearly polarized light at 0.6 THz, 0.65 THz and 0.7 THz, respectively. **(d)** The absolute efficiency of the meta-hologram based on full wave simulation. Here, we have respectively integrated the power passing through the imaging planes where two letters ("F" and "D") are shown and the power carried by the input linearly polarized light. The ratio between them is defined as the working efficiency of our meta-hologram.

H: Fabrication workflow of our high-AR dielectric metasurfaces

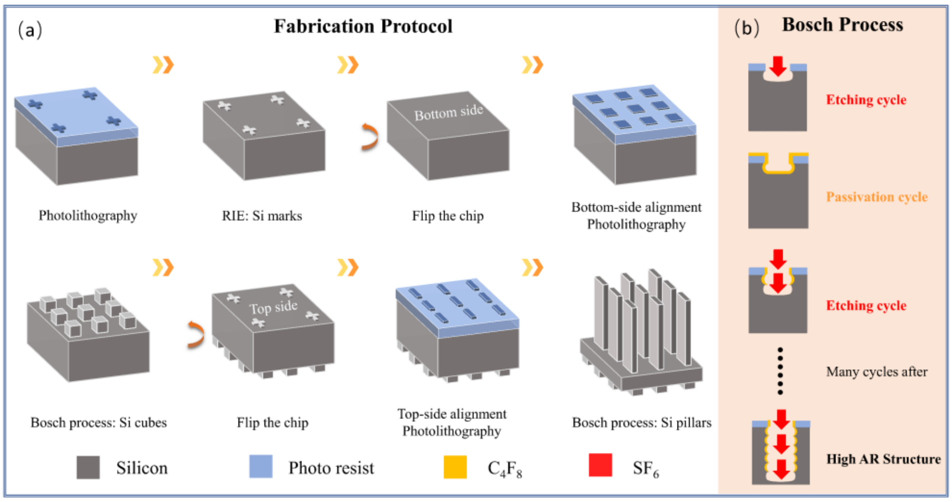

Figure S14. (a) Fabrication protocol for our dielectric metasurfaces. First, a spin-coating photoresist layer is covered on the top side of the silicon wafer. The photoresist layer is patterned with photolithography (Karl Suss MA6 Mask Aligner) and a set of silicon marks is transferred to the wafer using reactive ion etching. Next, the pattern of anti-reflection structures is transferred to the wafer through photolithography and etched by the advanced silicon etcher (Surface Technology System MUC-21) with a standard Bosch process. A final step of photolithography is performed on the top side of the wafer again. A high etch rate Bosch process recipe is employed in the last step to fabricate the high AR pillars of different feature sizes out of the wafer. **(b)** The Bosch process workflow: in the time-multiplexed deep etching technique, the etch active cycle and the passivation active cycle switch between each other in the process. The etch rate in horizontal direction is dramatically different from that in the vertical direction which makes the high AR etch realizable.

I: The state-of-the-art high-efficiency bifunctional metasurfaces

Table 1.State-of-the-art high-efficiency bifunctional metasuefaces

Literature	Frequency(or Wavelength)	Mode	Polarization	Material	Method	Efficiency (%)
[31]	10 GHz	Transmission	Circular	Metal	Expt	80
[26]	0.4 THz	Reflection	Circular	Metal	Expt	60
[46]	0.6 THz	Reflection	Circular	Metal	Expt	39
[33]	1 THz	Transmission	Circular	Silicon	Sim	60
[36]	1 THz	Transmission	Circular	Silicon	Sim	61
[47]	1 THz	Transmission	Linear	Silicon	Sim	65
[18]	860nm	Reflection	Circular	Metal	Expt	59
[48]	633nm	Transmission	Linear	Silicon	Expt	58
[29]	530nm	Transmission	Circular	TiO_2	Expt	54
[27]	325nm	Transmission	Circular	HfO_2	Expt	72
Our work	0.65 THz	Transmission	Circular	Silicon	Expt	**88**

*See References in the main text.
